# FOXM1 modulates docetaxel resistance in prostate cancer by regulating KIF20A

**DOI:** 10.1186/s12935-020-01631-y

**Published:** 2020-11-10

**Authors:** Hongbo Yu, Zheng Xu, Maomao Guo, Weiwan Wang, Weican Zhang, Sudong Liang, Zhibin Xu, Jun Ye, Gangyi Zhu, Chenyang Zhang, Jianzhong Lin

**Affiliations:** 1 Department of Urology, BenQ Medical Center, Nanjing Medical University, Nanjing, China; 2 Nanjing First Hospital, Nanjing Medical University, Nanjing, China; 3 Department of Urology, Hospital Affiliated 5 to Nantong University (Taizhou People’s Hospital), No. 366, Taihu Road, Taizhou, China; 4 Central Laboratory, BenQ Medical Center, Nanjing Medical University, Nanjing, China; 5 The First Clinical Medical College, Nanjing Medical University, Nanjing, China; 6 Central Laboratory, Hospital Affiliated 5 to Nantong University (Taizhou People’s Hospital), Taizhou, China

**Keywords:** FOXM1, Prostate cancer, Docetaxel, Resistance, KIF20A

## Abstract

**Background:**

Docetaxel resistance affects prognosis in advanced prostate cancer (PCa). The precise mechanisms remain unclear. Transcription factor Forkhead box M1 (FOXM1), which participates in cell proliferation and cell cycle progression, has been reported to affect the sensitivity of chemotherapy. This study explores the role of FOXM1 in PCa docetaxel resistance and its association with kinesin family member 20 A (KIF20A), which is known to promote therapeutic resistance in some cancers.

**Methods:**

We monitored cell growth using MTT and colony formation assays, and cell apoptosis and cell cycle progression using flow cytometry. Wound-healing and transwell assays were used to detect cell invasion and migration. mRNA and protein expression were analyzed using quantitative reverse transcription polymerase chain reaction (qRT-PCR) and western blotting. We monitored FOXM1 binding to the KIF20A promoter using a ChIP assay. Tumorigenicity in nude mice was used to assess in vivo tumorigenicity.

**Results:**

FOXM1 knockdown induced cell apoptosis and G2/M cell cycle arrest, suppressing cell migration and invasion in docetaxel-resistant PCa cell lines (DU145-DR and VCaP-DR). Exogenous FOXM1 overexpression was found in their parental cells. Specific FOXM1 inhibitor thiostrepton significantly weakened docetaxel resistance in vitro and in vivo. We also found that FOXM1 and KIF20A exhibited consistent and highly correlated overexpression in PCa cells and tissues. FOXM1 also regulated KIF20A expression at the transcriptional level by acting directly on a Forkhead response element (FHRE) in its promoter. KIF20A overexpression could partially reverse the effect on cell proliferation, cell cycle proteins (cyclinA2, cyclinD1 and cyclinE1) and apoptosis protein (bcl-2 and PARP) of FOXM1 depletion.

**Conclusions:**

Our findings indicate that highly expressed FOXM1 may help promote docetaxel resistance by inducing KIF20A expression, providing insight into novel chemotherapeutic strategies for combatting PCa docetaxel resistance.

## Background

Prostate cancer (PCa) is the second commonest cancer and a leading cause of male cancer deaths globally [1]. Chemotherapy using docetaxel remains the current modality of therapy for hormone-refractory and metastatic PCa. However, docetaxel resistance often leads to therapeutic failure and poor outcomes. Accumulating evidence suggests combining docetaxel with a targeted therapy that complements its mechanism of action can potentially delay the onset of resistance [2, 3]. Thus, identifying new therapeutic targets involved in PCa cell proliferation and metastasis is a key research objective.

FOXM1, a transcription factor mainly expressed in proliferative cells, is part of the Forkhead box family of transcription factors. Recent studies indicate FOXM1 is commonly overexpressed in many kinds of cancers, including PCa, and its expression is highly correlated with cancer proliferation and metastasis [4–8]. Several studies suggest FOXM1 overexpression confers acquired tolerance to chemotherapy through the regulation of numerous genes, including ATP-binding cassette genes [9, 10], DNA damage repair genes [11], apoptosis-associated genes [12], cancer stem cell-related genes [13, 14]. Previously, we showed that FOXM1 overexpression could reduce significantly the inhibitory effect of docetaxel on cell proliferation by inducing autophagy in PCa [15]. Unfortunately, the function and mechanisms-of-action of expressed FOXM1 during docetaxel resistance in PCa are still largely unknown.

Kinesin family member 20A (KIF20A) is believed to modulate microtubule dynamics, the target of taxanes. Several studies indicate KIF20A is transcriptionally regulated by FOXM1 in certain cancer cells, and that their expression is consistently elevated after treatment with paclitaxel. FOXM1 or KIF20A silencing significantly improved the chemosensitivity of paclitaxel [16, 17]. However, their potential interaction and effect on docetaxel-mediated PCa chemotherapy have yet to be explored. In this study, we invetsigated the effect of FOXM1 expression on apoptosis, cycle distribution, and the metastasis of PCa cells, examining whether FOXM1 downregulation increased cell sensitivity to docetaxel in vitro and in vivo. Since FOXM1 and KIF20A are consistently over-expressed in docetaxel-resistant PCa cells and tumor tissues, we explored whether FOXM1 contributed to docetaxel resistance by regulating KIF20A transcription and expression.

## Materials and methods

### Cell lines and cultures

Prostate cancer cell lines DU145 and VCaP were obtained from the Chinese Academy of Sciences, Shanghai Institute of Biochemistry and Cell Biology (Shanghai, China). DU145 cells were kept at 37 °C with 5% CO_2_ in RPMI 1640 media. VCaP cells were kept at 37 °C with 5% CO_2_ in DMEM medium. Media contained 10% fetal bovine serum and 1% streptomycin/penicillin.

Docetaxel-resistant cell lines DU145-DR and VCaP-DR were established as described previously [18]. Briefly, resistant PCa cells (DU145-DR and VCaP-DR) were generated by continuously exposing cells to increasing concentrations of docetaxel: 2 nM to 100 nM (DU145-DR) or 2 nM to 60 nM (VCaP-DR), over a 10-month period.

### Transfection

Sequences corresponding to FOXM1 and KIF20A siRNAs were: 5′-CUCUUCUCCCUCAGAUAUATT-3′ (FOXM1 siRNAs; sense sequence), 5′-UAUAUGAGGGAGAGTT-3′ (FOXM1 siRNAs; antisense sequence), 5′-GCAGCAGGUUCCAUCUGAGTT-3′ (siRNA KIF20A; sense), 5′-CUCAGAUGGAACCUGCUGCTT-3′ (siRNA KIF20A; antisense). The non‐targeting siRNA control sequence was 5′-UUCUCCGAACGUGUCACGUTT-3′. siRNAs were transfected using lipofectamine 2000 (Invitrogen, Carlsbad, CA, USA). pcDNA3.1/FOXM1 (pcFOXM1) and pc DNA3.1/KIF20A(pcKIF20A) plasmids were constructed using standard cloning methods. Cells were transiently transfected with pcFOXM1, pcKIF20A, and their NC plasmids, using lipofectamine 2000 (Invitrogen, Carlsbad, CA, USA). Mock-transfected cells served as a control (Ctrl). Transfection efficiency was evaluated after 48 h.

### Stable transfection

Human lentivirus-shFOXM1 was obtained from GenePharma (Shanghai, China). Lentiviruses were ultracentrifuged, concentrated, validated, and added to the culture medium. After infection, transduced cells were selected using puromycin (Gibco, Grand Island, NY, USA) over 2–3 weeks, and the surviving cells were continuously cultured. The resulting cell was referred to as DU145-DR stably transfected siFOXM1.

### Cell apoptosis analysis

Cells in an exponential growth phase were harvested and then seeded in six-well plates. Once cells became adherent, different treatments were undertaken. After cells had been cultured for 72 h, an Annexin V-FITC/PI Apoptosis Assay Kit was used to assess the percentage of apoptotic cells using BD FACS Calibur flow cytometry (BD Biosciences). The experiments were performed in triplicate.

### Cell cycle analysis

After treatment, cells were harvested and fixed for at least 4 h using 70% precooled ethanol. Cells were then incubated in PBS solution containing 50 mg/ml propidium iodide (PI) and 100 mg/ml RNase for 30 min at room temperature. Finally, the cell cycle status was analyzed using BD FACS Calibur flow cytometry (BD Biosciences, San Jose, CA), within 1 h.

### Cell viability assay and colony formation assay

Cells were seeded into a 96-well plate at 5 × 10^3^ cells/well and then treated. MTT (3-(4,5-dimethyl-2-thiazolyl)-2,5-diphenyl-2H-tetrazolium bromide) dye solution was added to each well and incubated for 4 h at 37 °C. The medium was aspirated and 100 μL of DMSO added to halt the reaction. Cell viability was measured spectrophotometerically using absorbance at 590 nm. For the colony formation assay, 500 cells were added to 6-well plates and cultured at 37 °C for 14 days. Colonies were staining with 0.1% crystal violet in methanol for 30 min. The number of colonies per well was then counted and analyzed.

### Wound-healing assay

Cells in the exponential growth phase were added to 6-well culture plates. After reaching confluence, the cell layer was scratched with a sterile P-200 pipette tip. The cells were then treated with docetaxel for 24 h. Photographs were taken (100 × magnification), and cell migration distances measured.

### Cell invasion

Cell invasion assays were undertaken using a modified transwell chamber with a Matrigel-coated membrane (BD Biosciences Bedford, MA), as described previously. Briefly, approximately 1 × 10^4^ cells were re-suspended in serum-free medium, and then plated onto 24-well plates. Re-suspended cells were treated with docetaxel, FOXM1-siRNA, pc-FOXM1, KIF20A-siRNA, and pc-KIF20A. At 24 h post-treatment, cell invasion was measured, following manufacturer’s instructions.

### Quantitative real-time PCR (qRT-PCR)

RNA was extracted from cells using a TRIzol reagent, following manufacturer’s instructions. RNA samples were then reversed transcribed using a PrimerScript RT reagent kit (Vazyme, Nanjing, China). RT-PCR was performed with an Applied Biosystems 7500 RT-PCR system using a Power SYBR Green PCR Master Mix (Vazyme, Nanjing, China). Primer sequences used for the PCR amplification were:

5′-TCCTCCACCCCGAGCAA-3′ (FOXM1-sense);

5′-CGTGAGCCTCCAGGATTCAG-3′ (FOXM1-antisense);

5′-TGCTGTCCGATGACGATGTC-3′ (KIF20A-sense);

5′-AGGTTCTTGCGTACCACAGAC-3′ (KIF20A-antisense).

All experiments were performed at least three times.

### Mouse PCa xenograft model for in vivo study

All animal experiments were approved by the Hospital of Nanjing BenQ Medical Center Animal Care and Use Committee. Animal experiments were undertaken following the Guide for the Care and Use of Laboratory Animals, as published by the National Institutes of Health. Briefly, DU145-DR cells (5 × 10^6^/100 μL) were subcutaneously injected into the two flanks of 6-week old nude mice (BALB/C-nu/nu, SLAC Laboratory, Shanghai, China). When the dimensions of a mouse xenograft reached 100 mm^3^, the subject was administered docetaxel (10 mg/kg) by i.p. injection twice a week or thiostrepton (TST, 30 mg/kg) by i.p. injection every-other-day. The size of tumor xenografts and the corresponding mouse body mass were measured every 4 days. The nominal tumor volume was calculated thus: tumor volume (mm^3^) = π/6 × (length) × (width)^2^. After one month, mice were euthanized. Tumors were removed by dissection, weighed, and prepared for subsequent experiments.

### Luciferase reporter assay

VCaP-DR cells were co-transfected with human KIF20A luciferase reporter (WT or MUT), siFOXM1 RNA and pRL-SV40 (Promega) using the TransIT-X2 Dynamic Delivery System (Mirus, Madison, WI, USA). The firefly/Renilla luciferase activities were detected 48 h after transfection using the Dual-Glo Luciferase reporter assay system (Promega), following manufacturer’s instructions. Luminescence was then measured using a spectraMax id3 three-mode microplate reader (Molecular Devices, MD, USA).

### Chromatin immunoprecipitation (ChIP)-PCR

Treated VCaP-DR cells were cross-linked using 1% formaldehyde and then lysed. The DNA fragment was sonicated to shear a mean DNA fragment. Potential chromatin–protein complexes were isolated by shearing into short, mean-lengthed DNA fragments using sonication. The resulting complexes were incubated with FOXM1 antibodies overnight at 4 °C, with IgG as a negative control. Some lysate was used for input control. Immune complexes were harvested using beads, before crosslinks were broken and DNA fragments purified. FOXM binding to chromatin DNA was quantified using qRT-PCR. The primers used were: 5′-TTCCTTACGCGGATTGGTAG-3′ (KIF20A sense); 5′-AGCCGCAGAGCACAACTC-3′ (KIF20A anti-sense); 5′-CCGCCTCCCTCTTAGCATAA-3′ (control sense); and 5′-CAGGAAATTGCATCTCGGGG-3′ (control anti-sense).

### Statistical analyses

Results are presented as mean ± standard deviation (S.D.). All statistical analyses were undertaken using GraphPad Prism 5.2 software (GraphPad Software, San Diego, USA). Differences between groups were analyzed using one-way analysis of variance (ANOVA). When a comparison involved two groups only, the Student t-test was used. Survival was analyzed using Kaplan–Meier curves. A log-rank test was used to determine statistical significance. A threshold of ^*^P < 0.05 was set for statistical significant.

## Results

### FOXM1 regulates apoptosis and cell cycle in docetaxel-treated PCa cells

**Fig. 1:**
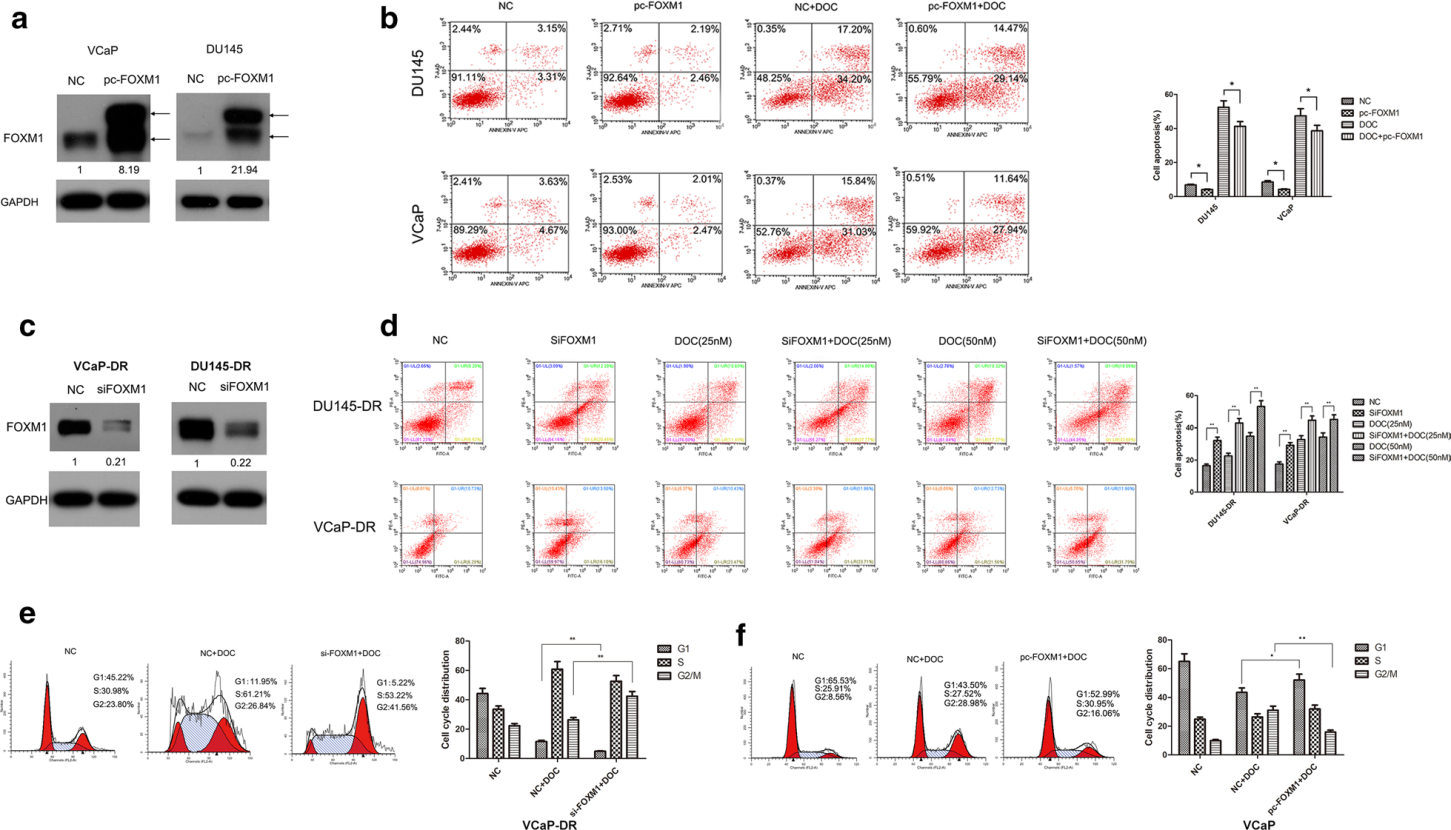
FOXM1 regulates apoptosis and cell cycle in docetaxel-treated PCa cells. **a** VCaP and DU145 cells were transfected with a FOXM1 plasmid, using lipofectamine 2000; western blotting (WB) confirmed overexpression of FOXM1 protein. **b** Cells were transfected with FOXM1 plasmid and treated with docetaxel (DOC, 25 nM). Apoptosis was detected using flow cytometry (FCM). **c** VCaP-DR and DU145-DR cells were transiently transfected with siRNA oligonucleotides targeting FOXM1 (siFOXM1) using lipofectamine 2000 and verified using WB. **d** FCM assessed cell apoptosis after FOXM1 knockdown alone or in combination with DOC (25 nM and 50 nM). **e** FOXM1 was depleted in VCaP-DR cells or in combination with DOC (25 nM). FCM was used to assess the cell cycle. **f** VCaP cells was transfected with pc-FOXM1 or combined with DOC (25 nM). Cell cycle distribution was determined by FCM. Data represent the mean ± SD of three independent experiments. ^*^p < 0.05, ^**^p < 0.01

We explored the functional relationship between FOXM1 expression and docetaxel resistance in PCa cells by first evaluating the influence of FOXM1 expression on apoptosis using flow cytometry (FCM). When a wild-type FOXM1 plasmid was transiently transfected for 72 h, FOXM1 overexpression in VCaP and DU145 cells was confirmed using WB (Fig. 1a). FCM results indicated that forced FOXM1 expression inhibited cell apoptosis significantly before and after docetaxel treatment when compared to non-transfected parental cells (Fig. 1b). Next, we investigated whether FOXM1 knockdown by siRNA enhanced docetaxel-induced apoptotic effects in docetaxel-resistant PCa cells. As shown in Fig. 1c, d, FOXM1 knockdown using RNA interference (siFOXM1) in docetaxel-resistant PCa cells, DU145/DR, and VCaP/DR, increased cell apoptosis significantly. FCM was also used to detect the relative cell cycle distribution between G1, S and G2/M phases after FOXM1 knockdown in docetaxel-treated VCaP-DR cells. This showed that combining siFOXM1 with docetaxel lowered G1 and enhanced G2/M phase ratios when compared to docetaxel treatment (Fig. 1e). The opposite was found for FOXM1 overexpression’s effect on the relative cell distributions in docetaxel-only treated cells (Fig. 1f). Together, this indicates FOXM1 regulates apoptosis and the cell cycle in docetaxel-treated PCa cells.

### FOXM1 expression is associated with docetaxel-induced inhibitory effects on PCa cells’ migration and invasiveness

**Fig. 2:**
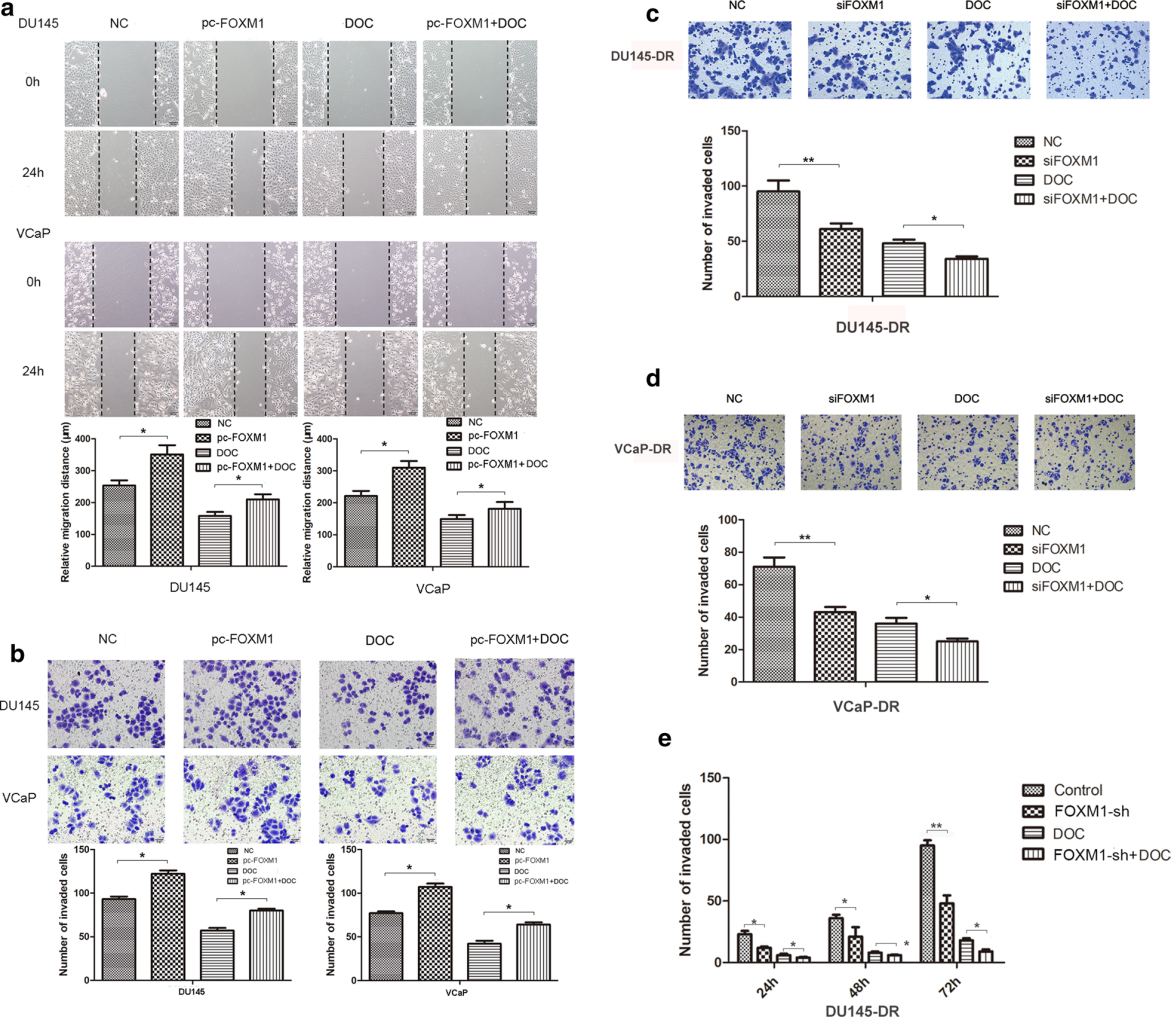
FOXM1 expression is closely associated with docetaxel-induced inhibition of migration and invasiveness in PCa cells. **a**, **b** VCaP and DU145 cells were transfected with FOXM1 plasmid or NC, using lipofectamine 2000 in 96-well plates. At 48 h post-transfection, cells were treated with 12.5 nM docetaxel (DOC) for 72 h. Wound closure assays and transwell invasion assays were undertaken to analyze migration and invasiveness of parental cells. **c**, **d** VCaP-DR and DU145-DR cells were treated with siFOXM1 and DOC (25 nM). Transwell invasion assays assessed the invasive capabilities of the cells. **e** Transwell invasion assays detected the invasive abilities of FOXM1 stable-knockout DU145-DR cells or in combination with DOC (25 nM) for 24 h, 48 h, and 72 h. Data represent the mean ± SD of three independent experiments. ^*^p < 0.05, ^**^p < 0.01

To investigate how FOXM1 expression affects PCa cells’ migration and invasiveness, we overexpressed FOXM1 in DU145 and VCaP cells and performed wound healing and transwell assays. Exogenous overexpression of FOXM1 enhanced the migratory and invasive capabilities of the cells significantly, blunting the inhibitory effect of docetaxel (Fig. 2a, b). Next, we investigated how invasiveness was affected by FOXM1 knockdown in DU145/DR and VCaP/DR cells, finding that the number of invading cells was decreased significantly by FOXM1-depletion in docetaxel-resistant cells. Combining FOXM1 knockdown and docetaxel caused a significantly stronger inhibition when compared to single treatment (Fig. 2c, d). We also analyzed the effect of FOXM1-mediated inhibition on the invasiveness of a stable FOXM1-knockdown DU145-DR cell line. Our results indicated FOXM1 inhibition decreased the invasiveness of stable DU145-DR cells significantly, enhancing docetaxel’s inhibitory effect in a time-dependent manner. Collectively, FOXM1 expression is related inversely to docetaxel’s effect on PCa cells’s migration and invasiveness.

### FOXM1 inhibition contributes to docetaxel induced suppression of proliferation in vitro and in vivo

**Fig. 3:**
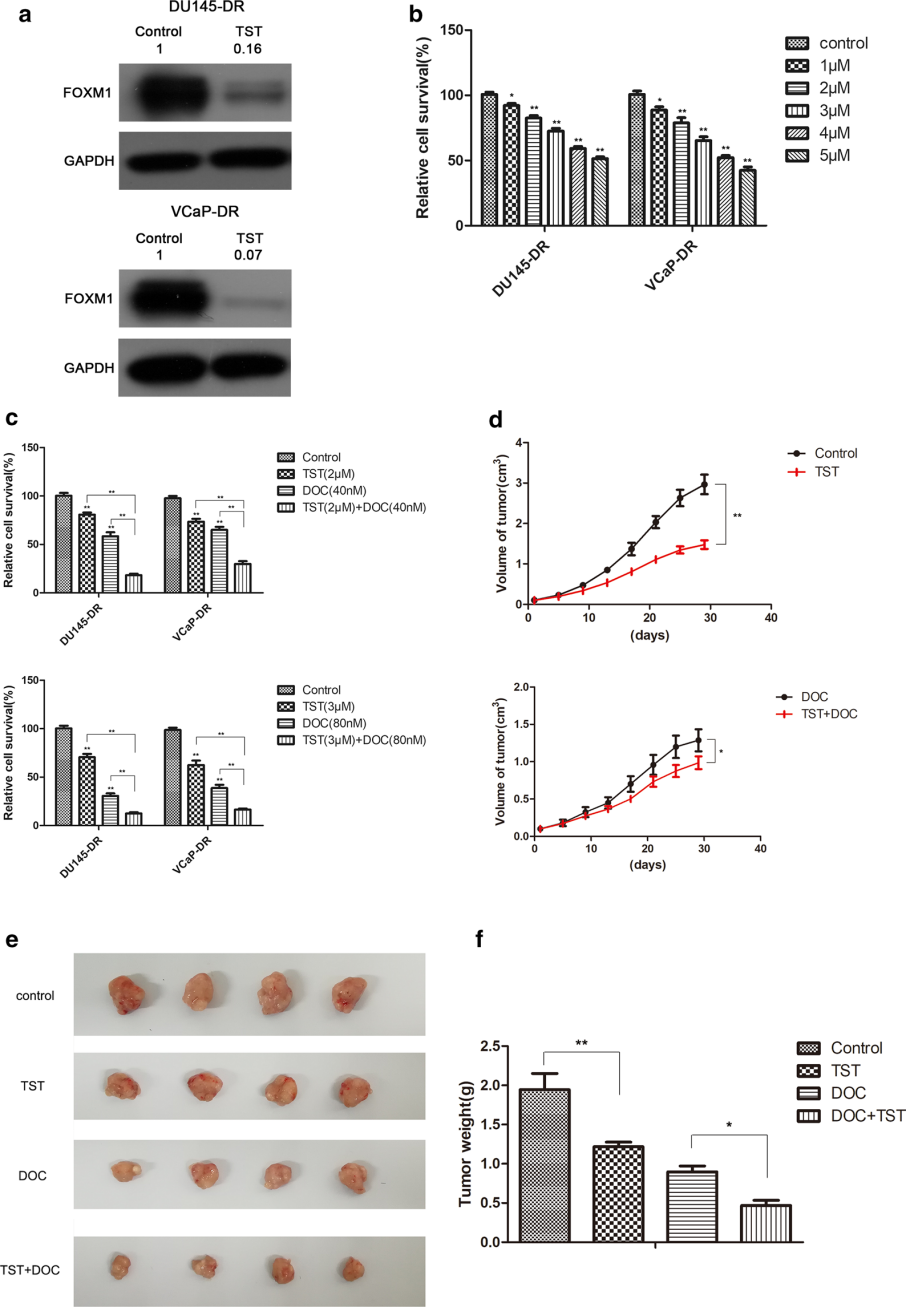
FOXM1 inhibition contributes to docetaxel sensitiveness in vitro and in vivo. **a** VCaP-DR and DU145-DR cells was treated with TST (3 μM). WB was used to analyze FOXM1 protein levels. **b** MTT was used to assess cell proliferation after administration of different thiostrepton (TST) doses (1 μM, 2 μM, 3 μM, 4 μM, 5 μM) for 72 h in VCaP-DR and DU145-DR cells. **c** Resistant cells were treated with different doses of thiostrepton (TST), docetaxel (DOC), or their combination. Cell viability was detected using MTT. D-F: Tumor xenografts were established in immunodeficient nude mice injected with DU145-DR cells. Nude mice with tumor xenografts were treated with or without DOC (10 mg/kg) by i.p. injection twice a week or TST (30 mg/kg) by i.p. injection every-other-day. The xenograft tumor size was measured every four days. **d** The tumor masses were harvested from immunodeficient nude mice at the end of the experiments. **e** The weight of tumors was measured and averaged for comparison between groups. **f** Data represents the mean ± SD (n ≥ 3). ^*^p < 0.05, ^**^p < 0.01

TST inhibited FOXM1 expression in DU145/DR and VCaP/DR cells (Fig. 3a) resulting in the retardation of proliferation of both resistant cell-lines in a dose-dependent manner (Fig. 3b). We also found that a combination of TST (2 μM and 3 μM) and docetaxel (40 nM and 80 nM) induced a stronger inhibition of proliferation than single treatment (Fig. 3c). We also explored if FOXM1 and TST reversed docetaxel resistance in vivo. Our results indicated TST treatment led to a significant inhibition of growth and a marked enhancement of the inhibitory action of docetaxel on xenograft tumors (Fig. 3d). Tumors were harvested from mice a month after the injection of cells. The size and average mass of tumors were less in xenografts treated with a combination of TST and docetaxel, than either single treatment group (Fig. 3e, f). TST-induced FOXM1 suppression appears to promote the chemotherapeutic effect of docetaxel in vitro and in vivo.

### FOXM1 and KIF20A are consistently expressed in PCa

**Fig. 4:**
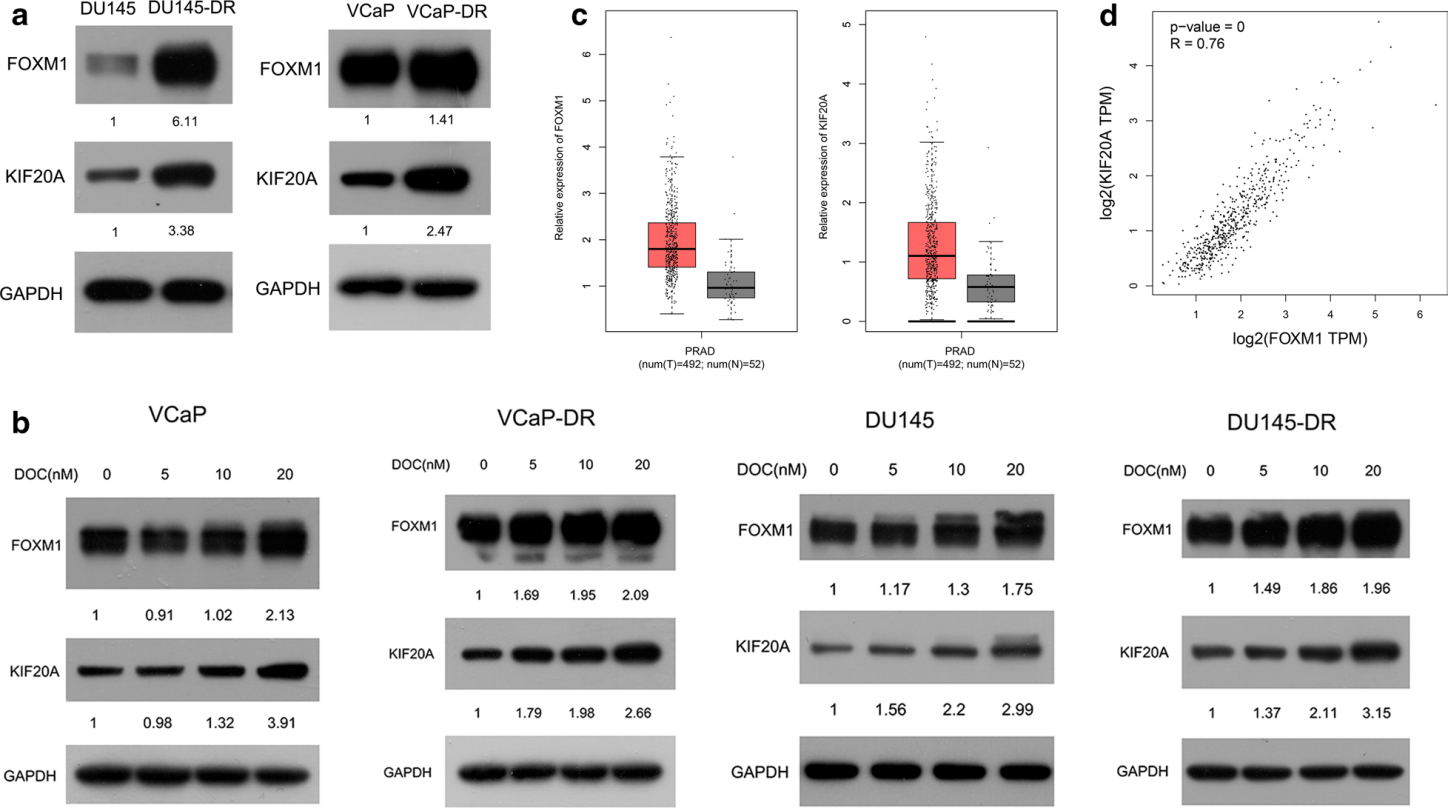
FOXM1 and KIF20A are consistently expressed in PCa. **a** Protein expression of FOXM1 and KIF20A in resistant and parental cells was analyzed using WB. **b** VCaP, DU145, and their respective resistant cell variants were treated with different doses of DOC (0 μM, 5 μM, 10 μM, 20 μM). WB was performed to assess protein levels of FOXM1 and KIF20A in the presence and absence of DOC. Quantitative analysis was performed using the Gel-Pro 32 software. **c** FOXM1 and KIF20A mRNA expression levels were analyzed in either 492 prostate cancer tumor tissues or 52 normal prostate tissues using TCGA data. **d** Correlation analysis was used to analyze the correlation in expression of FOXM1 and KIF20A in prostate cancer tissues in the TCGA database. ^*^p < 0.05

It is well known that KIF20A is abnormally highly expressed in many tumors and is associated with a poor prognosis, including for PCa [16, 17, 19]. The association between FOXM1 and KIF20A in PCa was therefore investigated, with the protein levels of FOXM1 and KIF20A in DU145, VCaP, and their resistant cells being assessed. We found FOXM1 and KIF20A were expressed at much higher levels in docetaxel-resistant cells than in their parent cells (Fig. 4a). Expression of KIF20A had similar kinetics to FOXM1 with different doses of docetaxel (Fig. 4b). We also analyzed FOXM1 and KIF20A expression levels using TCGA data. This indicated both FOXM1 and KIF20A mRNA levels were increased significantly in PCa (Fig. 4c). Statistical analysis demonstrated a high correlation between FOXM1 and KIF20A expression in PCa (R = 0.76) (Fig. 4d). Such data shows FOXM1 and KIF20A expression is highly correlated, giving rise to drug resistance and a poor prognosis in PCa.

### FOXM1 regulates KIF20A gene transcription in PCa.

**Fig. 5:**
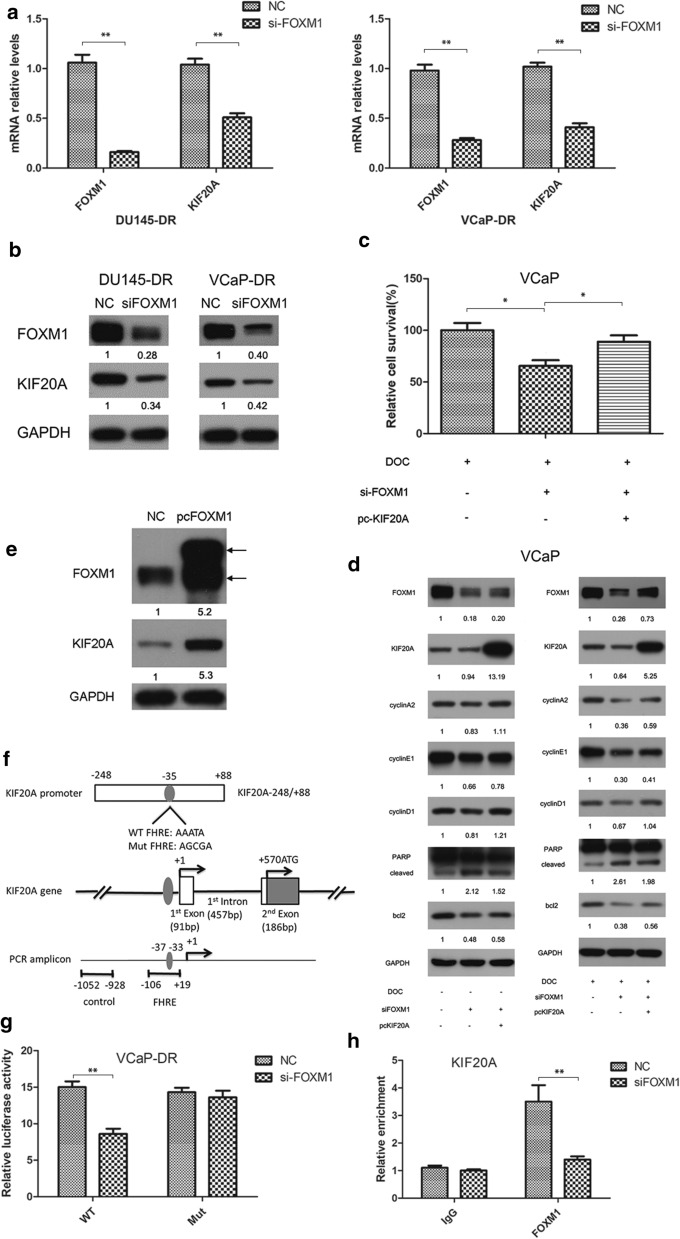
FOXM1 regulates KIF20A gene transcription in PCa. **a**, **b** FOXM1 expression was knocked down in VCaP-DR and DU145-DR cells using siRNA. qRT-PCR and WB were used to measure KIF20A mRNA and protein levels. **c**, **d** VCaP cells were transfected with siFOXM1 or in combination with pc-KIF20A. MTT was used to assess cell proliferation. WB was used to analyze levels of cyclinA2, cyclinD1, cyclinE1, bcl-2, and PARP protein in the absence or presence of DOC (12.5 μM). **e** FOXM1 expression was upregulated by pc-FOXM1. KIF20A was then detected using WB. **f**–**h** FOXM1 knockdown in VCaP-DR cells decreased KIF20A gene promoter activity. The FOXM1 protein-bound DNA in VCaP cells was purified using ChIP. Primers spanning the FKH binding motif of the KIF20A gene promoter were designed. FOXM1 binding to the human KIF20A promoter was examined using ChIP-PCR. Protein quantitative analysis was undertaken using the Gel-Pro 32 software, Data represents the mean ± SD (n = 3). ^*^p < 0.05, ^**^p < 0.01

Since FOXM1 and KIF20A were consistently expressed in PCa cells, we investigated if FOXM1 regulated KIF20A, and found FOXM1-depletion decreased KIF20A at the mRNA and protein levels in VCaP-DR and DU145-DR cells (Fig. 5a, b). To evaluate the regulatory effect of FOXM1 on KIF20A during docetaxel chemotherapy, siFOXM1 for FOXM1 inhibition and pcKIF20A for KIF20A overexpression were transfected into VCaP cells, which were then treated with docetaxel. MTT results indicated that overexpression of KIF20A reduced siFOXM1-mediated inhibition of proliferation (Fig. 5c). siFOXM1 was found to decrease levels of cyclinA2, cyclinD1, cyclinE1, bcl-2 and PARP, which are thought to be associated with the chemotherapeutic effect of docetaxel. Conversely, KIF20A overexpression could significantly reverse the siFOXM1-induced decrease in their expression (Fig. 5d). These regulatory effects also existed in the absence of docetaxel treatment (Fig. 5d). Moreover, the forced overexpression of FOXM1 upregulated KIF20A protein expression in VCaP cells (Fig. 5e). We conclude from these results that FOXM1 regulates expression of the KIF20A gene.

To clarify the mechanisms underlying FOXM1 regulation of KIF20A gene transcription, the influence of FOXM1 on the promoter activity of KIF20A was assessed using a luciferase reporter assay. We found siFOXM1 significantly decreased KIF20A gene promoter activity (Fig. 5f, g). We also tested FOXM1 binding to the KIF20A gene promoter using ChIP-qPCR. As shown in Fig. 5h, ChIP analysis indicated that siRNA inhibition significantly decreased FOXM1 binding to the KIF20A promoter region. Overall, our results suggest FOXM1 regulates KIF20A gene transcription by binding directly to the FHK motif in its promoter.

### The depletion of KIF20A sensitized the resistant cells to docetaxel in PCa

**Fig. 6:**
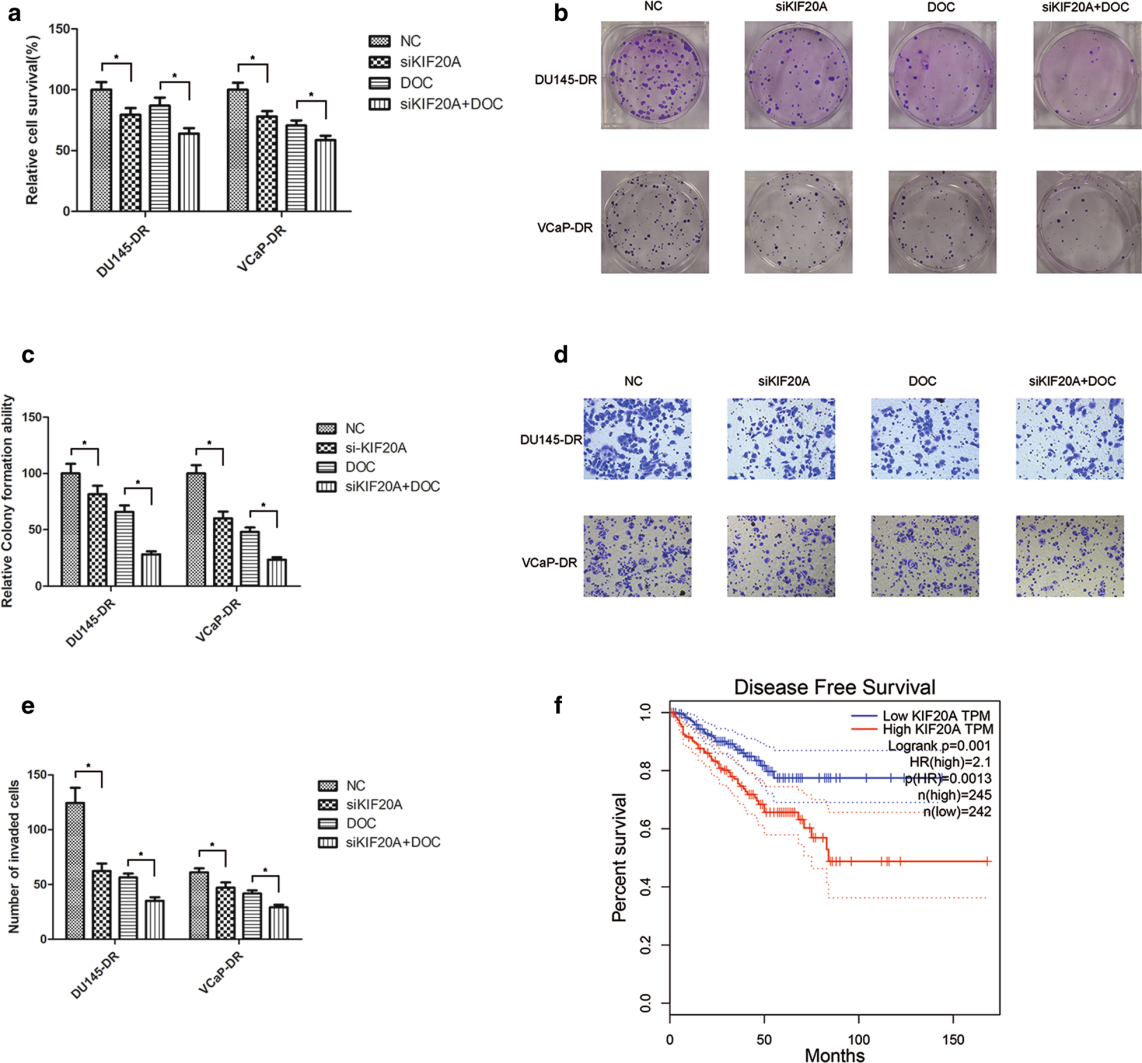
The depletion of KIF20A sensitized the resistant cells to docetaxel. VCaP-DR and DU145-DR cells were treated with siKIF20A and then DOC (25 nM). After administering DOC for 72 h, an MTT assay (**a**) or 14 days in a colony formation assay (**b**, **c**) were used to assess the viability of cells. **d**, **e** The invasive abilities of cells were assessed using a transwell invasion assay. **f** Kaplan–Meier survival analysis indicates that the association of KIF20A expression with poorer disease free survival was statistically significant. Data represents the mean ± SD of three experiments. ^*^p < 0.05, ^**^p < 0.01

Cell proliferation was assessed by depleting KIF20A in DU145-DR and VCaP-DR cell lines using siRNAs, indicating that KIF20A knockdown promoted docetaxel’s inhibitory effect on the proliferation of resistant cells, as suggested by using MTT (Fig. 6a) and clone (Fig. 6b, c) assays. KIF20A depletion also greatly enhanced docetaxel’s inhibition of the invasion of resistant cells (Fig. 6d, e). Survival analysis indicated that high KIF20A expression was significantly associated with poor disease-free survival, based on TCGA data (P < 0.01) (Fig. 6f).

## Discussion

Docetaxel is regarded as first-line chemotherapy, exerting anti-tumor efficacy in advanced PCa. However, drug resistance occurs after prolonged use. Delaying resistance to docetaxel may improve the therapeutic outcome for PCa patients. Several studies indicate that development of docetaxel resistance is associated with FOXM1upregulation, and can be reversed by the down-regulation of FOXM1 expression in some cancers [12, 20, 21]. High FOXM1 expression is also associated with the late stages of PCa, high gleason score, and poor prognosis [22]. In this study, we found FOXM1 levels increased significantly with the development of acquired resistance in PCa. Our study also suggests treatment with docetaxel led to an upregulation of FOXM1. Together, this indicates FOXM1 expression may affect the therapeutic efficacy of docetaxel in advanced PCa. However, the association of FOXM1 expression with PCa chemotherapeutic clinical characteristics needs to be confirmed by future studies with larger patient cohorts.

It is well known that PCa undergoes cellular dormancy as it becomes drug resistant, resulting in cell cycle arrest and formation of lethal metastatic disease. FOXM1 is a cell growth-specific transcription factor with a crucial role in regulating the transition from the G1 to S phase and in controlling G2/M cell cycle progression [23–25]. Overexpression of FOXM1 contributes to uncontrolled cell division and an unstable cellular microenvironment, while silencing it enhances apoptosis and increases sensitivity to chemotherapy [26–28]. Consistent with other studies, we showed that FOXM1 helps regulate the cell cycle and that depleting it during docetaxel treatment increases apoptosis in PCa cells. Reducing FOXM1 expression can lead to decreased cancer progression and an increased sensitivity to chemotherapy in many tumors. Thiostrepton (TST), a cyclic oligopeptide antibiotic FOXM1 inhibitor, has been shown to enhance cancer cell chemo-sensitization significantly, leading to reduced proliferation [29, 30]. Our data indicated FOXM1 inhibition by TST in resistant PCa cells strongly suppressed cell proliferation, promoting sensitivity to docetaxel-induced cytotoxicity. A PCa xenograft mouse model suggested that TST consistently inhibited tumor growth, enhancing docetaxel sensitivity. Our results suggest inhibition of FOXM1 expression can reverse docetaxel resistance in PCa, while strengthening the effects of docetaxel in chemotherapy.

Although docetaxel treatment improves overall outcomes for PCa, ultimate mortality in metastatic PCa is still high due to acquired resistance developed through many cycles of docetaxel therapy. FOXM1 has been identified as a master regulator of tumor invasion and metastasis [8, 31–33]. Our results indicated that overexpression of FOXM1 in parental PCa cells counteracted docetaxel’s inhibition of migration and invasion capacities of parental cells. Moreover, docetaxel resistance in invading cells could be partially reversed by siRNA-mediated FOXM1 inhibition. These observations are consistent with previous studies, indicating that overexpression or downregulation of FOXM1 significantly modulated the effects on cell migration and invasion of many chemotherapeutic drugs [9–13]. Overall, this suggests the clear oncogenic role of FOXM1 in maintaining tumor invasion and metastasis.

Upregulation of KIF20A gene is known to be associated with increased resistance to multiple chemotherapeutic agents [16, 17, 34, 35]. KIF20A has been shown to promote the tumorigenesis and progression of PCa, especially its biochemical recurrence and metastasis [36]. Yet, KIF20A’s role in docetaxel-resistant PCa remains poorly understood. We argue here that KIF20A may be another important regulator for promotion of docetaxel resistance in PCa. We observed that KIF20A expression, like FOXM1, was clearly greater in PCa resistant cells than in parental cells. Downregulation of KIF20A by siRNA, sensitized resistant PCa cells to docetaxel-induced cell killing. This is indicated by artificially suppressing proliferation and invasion, and by increasing docetaxel-induced inhibition of these effects. Statistical analysis of TCGA data also indicates clearly that KIF20A overexpression associates with poorer patient survival. These results suggest expression of KIF20A may alter the chemo-sensitivity and prognosis of PCa.

Previous studies have reported that FOXM1 and KIF20A levels are correlated in many tumors [16, 17, 37]. Our results suggest that docetaxel upregulated FOXM1 and KIF20A expression, while FOXM1 depletion decreased KIF20A expression in PCa resistant cells, which indicates KIF20A may be a downstream target of FOXM1in PCa. TCGA data analysis indicates a significant correlation between FOXM1 and KIF20A expression in PCa. To explore the relationship between FOXM1 and KIF20A, ChIP-PCR and a luciferase reporter assay were undertaken. They suggested that FOXM1 drove transcription of KIF20A at the promoter level through a forkhead response element, further strengthening our assertion that FOXM1 directly regulates KIF20A in PCa.

KIF20A has been reported to be implicated in FOXM1-related chemoresistance in hepatocellular carcinoma and breast cancer [16, 17]. In our study, we explored if FOXM1 could alter the sensitivity to docetaxel of PCa cells through KIF20A. We found overexpression of KIF20A reversed FOXM1 inhibition of the cell-killing effect of docetaxel in PCa. Increasing evidence indicates cyclin A2, cyclin D1 and cyclin E1 are positively correlated with the development of chemotherapy resistance, while the knockdown or depletion of these proteins inhibits cancer cell proliferation and promotes apoptosis, increasing sensitivity to chemotherapeutic drugs [38–40]. Our data revealed that depletion of FOXM1 in PCa cells downregulated these proteins, while KIF20A upregulation partially restored their expression. A similar effect was also seen for the regulation of apoptosis-related proteins bcl-2 and PARP.

Our research greatly extends previous work and gives support to the concept that FOXM1 mediates KIF20A regulation of activity in docetaxel-resistant PCa cells. However, whether KIF20A has a reciprocal effect on FOXM1 remains an open question in need of urgent future research.

The present study has demonstrated that increased FOXM1 expression is implicated in docetaxel resistance in PCa. We have also outlined a novel mechanism by which FOXM1 regulates docetaxel resistance in PCa cells mediated by KIF20A. This study also validates the conjecture that a FOXM1-KIF20A axis could serve as a potential target for therapy able to subvert docetaxel resistance in patients with advanced PCa.

## Data Availability

The datasets used and analyzed during the current study are available from the corresponding author on reasonable request.

## Abbreviations

PCa: Prostate cancer
FOXM1: Forkhead box M1
KIF20A: Kinesin family member 20 A
ATP: Adenosine triphosphate
FCM: Flow cytometry
TST: Thiostrepton
MTT: 3-(4,5-Dimethyl-2-thiazolyl)-2,5-diphenyl-2H-tetrazolium bromide
qRT-PCR: Quantitative reverse transcription polymerase chain reaction
NC: Negative control
CHIP: Chromatin immunoprecipitation
